# Super-resolution of fluorescence-free plasmonic nanoparticles using enhanced dark-field illumination based on wavelength-modulation

**DOI:** 10.1038/srep11447

**Published:** 2015-06-15

**Authors:** Peng Zhang, Seungah Lee, Hyunung Yu, Ning Fang, Seong Ho Kang

**Affiliations:** 1 Department of Chemistry, Graduate School, Kyung Hee University, Yongin-si, Gyeonggi-do 446-701, Korea; 2 Department of Applied Chemistry and Institute of Natural Sciences, Kyung Hee University, Yongin-si, Gyeonggi-do 446-701, Korea; 3 Center for Nanometrology, Korea Research Institute of Standards and Science, Daejeon 305-340, Korea; 4 Ames Laboratory-US Department of Energy and Department of Chemistry, Iowa State University, Ames, Iowa 50011, USA

## Abstract

Super-resolution imaging of fluorescence-free plasmonic nanoparticles (NPs) was achieved using enhanced dark-field (EDF) illumination based on wavelength-modulation. Indistinguishable adjacent EDF images of 103-nm gold nanoparticles (GNPs), 40-nm gold nanorods (GNRs), and 80-nm silver nanoparticles (SNPs) were modulated at their wavelengths of specific localized surface plasmon scattering. The coordinates (*x, y*) of each NP were resolved by fitting their point spread functions with a two-dimensional Gaussian. The measured localization precisions of GNPs, GNRs, and SNPs were 2.5 nm, 5.0 nm, and 2.9 nm, respectively. From the resolved coordinates of NPs and the corresponding localization precisions, super-resolution images were reconstructed. Depending on the spontaneous polarization of GNR scattering, the orientation angle of GNRs in two-dimensions was resolved and provided more elaborate localization information. This novel fluorescence-free super-resolution method was applied to live HeLa cells to resolve NPs and provided remarkable sub-diffraction limit images.

Optical microscopy imaging is one of the most widely used techniques for biomedical and molecular biology research^1^. In contrast to other microscopic techniques such as electron microscopy, it shows high usability and feasibility for intravital detection^2^. However, until the development of super-resolution microscopy, conventional optical microscopy techniques were incapable of resolving sub-cellular structures smaller than one hundred nanometers due to the diffraction limitation of light; these structures include machinery/microtubules that are 25 nm in diameter, transport vesicles approximately 100 nm in size, and 30-nm-wide chromatin fibers^3^,^4^,^5^.

By modifying the point spread function (PSF) of emitters, stimulated emission depletion (STED)^6^, ground-state depletion (GSD)^7^, and saturated structured illumination microscopy (SSIM)^8^ have provided high-resolution images beyond the diffraction limit. On the other hand, with the benefit of single-molecule detection and localization, stochastic optical reconstruction microscopy (STORM) and photoactivated localization microscopy (PALM) can be used to resolve sub-cellular structures with better than 20-nm resolution^9^,^10^,^11^. Since single molecule detection based-techniques can be carried out with conventional fluorescence microscopes, they have been widely used in molecular biology and biophysics^12^,^13^,^14^,^15^. However, since these super-resolution optical microscopy techniques are highly dependent on photo-switchable fluorescent probes, the selection of adequate fluorescent probes has become one of the most critical challenges for researchers^13^,^16^,^17^. Even though many natural and synthetic fluorescent probes have been developed, most of them are still not “bright” enough, i.e., they have low extinction coefficients and quantum yields, are highly sensitive to the environment, and are susceptible to photobleaching^17^,^18^,^19^,^20^. Therefore, development of “bright”, stable, and non-bleaching labeling agents is an urgent challenge to expand applications of super-resolution methods in biological research^17^. To overcome this challenge, plasmonic nanoparticles (NPs) may serve as appropriate fluorescence-free labeling agents for super-resolution imaging. Plasmonic NPs such as gold nanoparticles (GNP), gold nanorods (GNR), and silver nanoparticles (SNP) have been used as labeling agents in biological and biomedical research due to their large contrast and refined optical scattering properties in the visible region of the spectrum^21^,^22^,^23^. So far, only the super-resolution of adjacent SNPs was achieved by deconvolution of their spectra^24^,^25^. Although recent research has reported sub-diffraction limited resolution of GNR based on their anisotropic scattering properties^26^, more general sphere plasmonic NPs could not be resolved using this methodology. Herein, we report a fluorescence-free super-resolution imaging method using EDF illumination based on wavelength modulation to resolve various single plasmonic NPs with sub-diffraction-limit resolution.

## Results

### Wavelength-modulation EDF imaging of single plasmonic NPs

**Figure 1:**
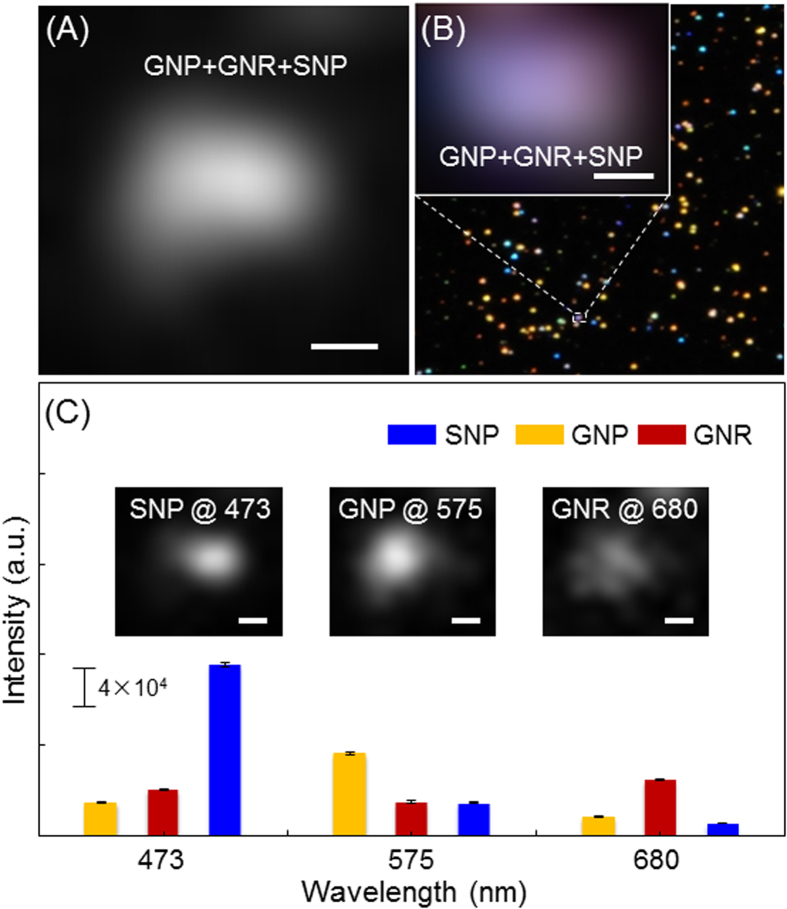
(**A**) EMCCD and (**B**) colored digital camera images of the mixture of GNPs, GNRs, and SNPs. (**C**) Scattering intensities of GNP, GNR, and SNP with various band-pass filters (473 ± 10 nm, 575 ± 15 nm, and 680 ± 10 nm) and the corresponding EDF images (inset). The scattering intensities of GNP, GNR, and SNP at 473 nm were 2979 ± 49.6, 4110.6 ± 63.7, and 15120.6 ± 155.0, at 575 nm were 7266 ± 91.1, 3021.8 ± 107.3, and 2924.2 ± 85.8, and at 680 nm were 1703 ± 30.3, 4975.6 ± 92.1, and 1126.8 ± 6.8, respectively. The EMCCD camera exposure time was 91 ms. The EMCCD gain was varied from 0 to 1000. The scale bars represent 200 nm. The error bars represent mean ± standard deviations (*n* = 5).

Far-field optical microscopy images of NPs are quite different from their true features (Supplementary Figs S1 and S2). Due to the diffraction limit, some intrinsic and specific details of these materials are lost, such as particle size and shape. Even worse, for NPs within a distance of 100 nanometers, recognition of these aspects from individual far-field microscopy images was impossible (Fig. 1A). Although the dark-field images provided specific color images depending on the material properties, which could help in the recognition of NPs, the cross-talk of scattered light from adjacent particles resulted in color-blended images (Fig. 1B and Supplementary, Fig. S3). However, these blurred and color-blended images were modulated with band-pass filters (Fig. 1C) based on the wavelength of the unique localized surface plasmon resonance (LSPR) scattering (Supplementary, Fig. S4). After modulation with band-pass filters, the cross-talk of scattering spectra was significantly suppressed (Fig. 1C). Therefore, the NPs could be detected at a distinct specific wavelength and resolved with sub-diffraction limit resolution.

### Gaussian fitting of the center of individual NPs

**Figure 2:**
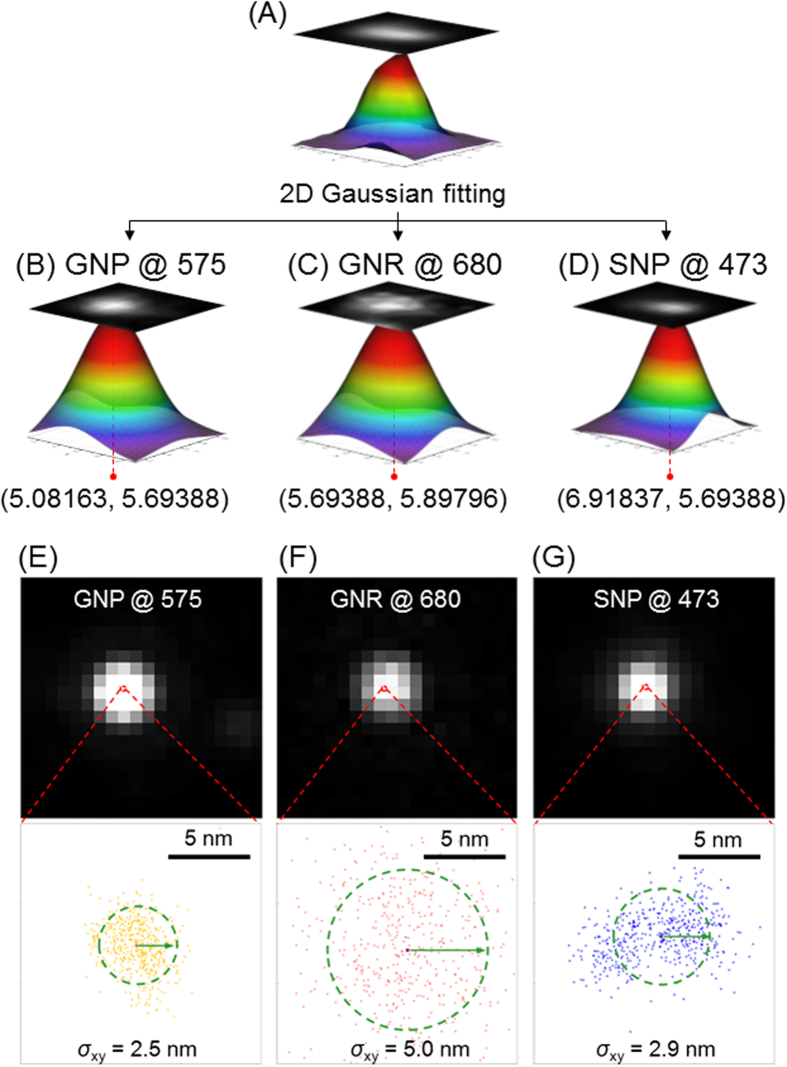
2D Gaussian fitting of wavelength-modulation images of GNPs, GNRs, and SNPs in a mixture sample at their specific scattering wavelengths to solve the center coordinates (*x, y*) of PSF. (**A**) Original blurred EDF image of adjacent GNPs, GNRs, and SNPs in the mixture sample, (**B**) wavelength-modulation EDF images of GNP at 575 nm, (**C**) GNR at 680 nm, and (**D**) SNP at 473 nm. The corresponding central coordinates after 2D Gaussian fitting were (5.05163, 5.69388), (5.69388, 5.89796), and (6.91837, 5.69388), respectively. (**E**, **F**, and **G**) Experimentally-recorded images of single NP illuminated by an EDF condenser with wavelength-modulation. The dots represent central position distributions of 499 measurements. *σ*_xy_ represents the lateral standard deviation.

When a point-like source is imaged, its projection on a detector is referred to as the PSF. For an isotropic point-like source, the PSF can be expressed as a Born-Wolf model with high precision and accuracy^27^. Even though several fitting/non-fitting-based PSF center localization algorithms have been reported, the least-squares criterion-based Gaussian fitting algorithm (Supplementary Fig. S5) is preferred due to its high localization precision, high fit speed, and need for few fit parameters^5^,^28^. The 2D Gaussian function for fitting the center of the PSF of each individual NP at the intrinsic scattering wavelength is given by the following equation^29^,^30^,^31^,^32^:


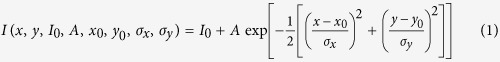


where *I*_0_ is a constant term from the background noise, *A* is the amplitude, *x*_0_ and *y*_0_ are the coordinates of the center, and *σ*_x_ and *σ*_y_ are standard deviations of the distribution in the *x* and *y* directions. The center coordinates (*x, y*) are recorded in Fig. 2B–D.

### Sub-diffraction localization precision analysis

In practice, users often determine the experimental localization precision in an experiment by measuring multiple localizations of the same single molecule and calculating the standard deviations (*SD*) of the Gaussian distributions of localization^17^,^29^. When calculating the lateral *SD*, the precision of localization values were as low as 2.9, 2.5, and 5.0 nm, respectively (Fig. 2E–G). Noticeably, the localization precision for GNR (5.0 nm) was much poorer than for GNP (2.5 nm) and SNP (2.9 nm). Depending on the single-particle localization theory, the best localization precision for an individual emitter in optical microscopy is determined by the root of the Cramér-Rao lower bound (CRLB), which is defined as the smallest possible variance any unbiased estimation algorithm can provide^29^,^33^,^34^. The CRLB is given as


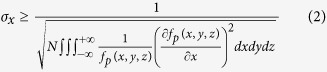


Considering the two-dimensional Gaussian approximation of PSF and only shot noise, the CRLB in the *x*-direction (*σ*_x_) can be simplified to


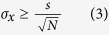


where *N* is the number of detected photons and *s* is the standard deviation of the Gaussian function. The CRLB in the *y*-direction (*σ*_y_) is a similar expression. The factors that can affect the detected photons, such as exposure time of the CCD camera, can affect the localization precision (Supplementary, Fig. S6). Interestingly, the localization precisions for GNR were much poorer than for GNP and SNP with any exposure time. According to Mie theory, the scattering cross-section of 40-nm GNR was much smaller than the 103-nm GNP and 80-nm SNP due to the size effect, which resulted in fewer detected photons (*N*) from GNR^35^,^36^. Therefore, the 40-nm GNR showed the poorest localization precision compared with the 103-nm GNP and 80-nm SNP. Due to the large scattering cross-sections and photo-stabilities of plasmonic NPs^21^, the localization precisions of all three types of NPs were better than most previously reported fluorescence-labeling super-resolution methods^5^,^32^.

### Reconstruction of the super-resolution image based on Gaussian rendering

**Figure 3:**
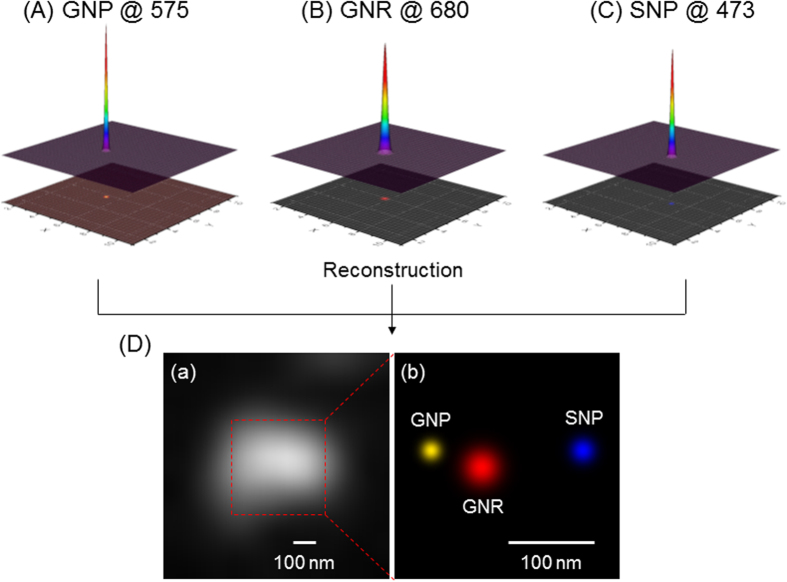
Fluorescence-free super-resolution images of GNP, GNR, and SNP in a mixture sample using EDF illumination based on wavelength-modulation. The pixel sizes of the rendered images of (**A**) GNP, (**B**) GNR, and (**C**) SNP were 2.5 nm, 5.0 nm, and 3.0 nm, respectively. The original image (**D**(**a**)) was reconstructed to form the super-resolution image (**D**(**b**)). Distances between particles in the reconstructed super-resolution image were 75 nm and 105 nm, respectively (**D**(**b**)).

With the measured localization precision, images (pixel matrix) of GNPs, SNPs, and GNRs were rendered as pixel sizes of 2.5 nm, 3.0 nm, and 5.0 nm, respectively. The rendered GNP, SNP, and GNR images were merged together to obtain the reconstructed image (Fig. 3D(b)). Compared with the blurred and unrecognizable original image (Fig. 3D(a)), the GNP, GNR, and SNP images were clearly resolved. The distance between the GNP and GNR was 75 nm, and the SNP was in the vicinity of the GNR with a distance of 105 nm. For adjacent GNP and SNP, the resolved distance was 105 nm. The value was comparable to the average size of the GNP and SNP, and it almost was the smallest distance to neglect the plasmonic coupling effect^26^. The measured distance of GNP and GNR was 75 nm which was less than the average size of two particles. In the case, the coupling of particles’ surface plasmon should be considered^37^. Due to the coupling of interparticles, the measured localization precisions of adjacent particles were lower than their free single-particle state (Supplementary, Fig. S7). However, the localization precision of GNP and GNR were still less than 15 nm. Therefore, even the measured distance value (75 nm) between GNP and GNR would contain the errors from plasmonic coupling effect, these errors should be tolerable. While, due to the plasmonic coupling effect of these 100-nm scale NPs, it would be difficult to achieve 10-nm resolution, which is comparable to recently reported fluorescence super-resolution microscopy^14^. In the future, to obtain better sub-diffraction limit resolution (i.e., less than 10 nm) for fluorescence-free detection would require much smaller size NPs (Supplementary, Fig. S8).

### Analysis of GNR orientation angle

For an anisotropic object such as a GNR, apart from its 2D coordinates (*x, y*), its rotation state significantly affects its optical properties and bio-activities^38^,^39^,^40^. In general, the nanorod in solution shows a free rotation state in 3D (Supplementary, Fig. S9(A))^38^. However, considering Supplementary Figs S1 and S2, most of the nanorods were “laying” on a 2D surface (i.e., polar angle *θ* = 90°). To simplify the model, the polar angle *θ* was assumed to be constant at 90°, and the rotation state of the GNR was therefore only related to its orientation angle, *φ*. Thus, a more precise localization expression of the GNR should include its orientation angle on the *x-y* plane (*φ*, Supplementary, Fig. S8(B)). According to Gans theory, scattered light from a gold nanorod has an inherent polarization^41^. Additionally, under homogeneous illumination conditions, the scattering intensity (*I*) of a gold nanorod in one particular polarization direction is proportional to the square of the cosine of the angle *φ* between the rod and the polarization direction^42^,^43^, as expressed in equation (4):


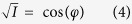


**Figure 4:**
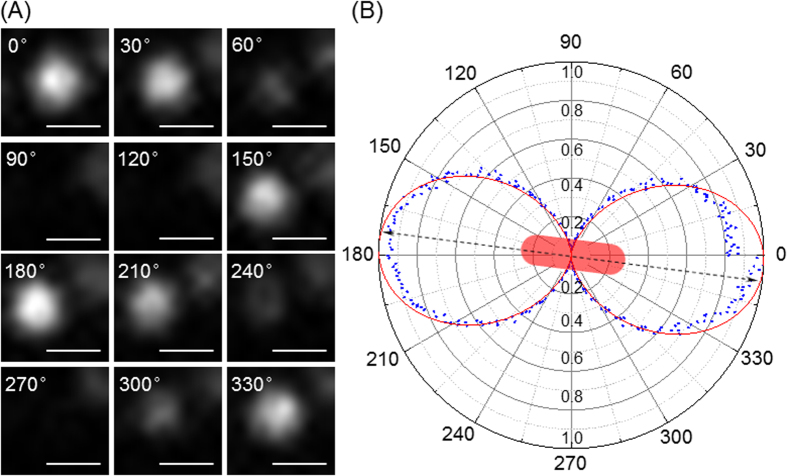
(**A**) The orientation angle dependent twinkle of GNR and (**B**) the root of normalized scattering intensity of GNR at 680 nm (*I*^0.5^) with various polarizer directions plotted in polar coordinates. The orientation angle relative to the polarizer direction was 173°. The scale bars represent 100 nm.

This induces a polarization based twinkle for GNR (Fig. 4(A) and Movie S1). Therefore, the rotation angle is resolved from its polarized scattering intensity profile. Herein, the scattering intensity of GNR at its specific wavelength was measured with various polarization angles and was plotted in polar coordinates (Fig. 4B). This plot indicated the orientation angle of the GNP was 173° relative to the polarizer direction. Furthermore, due to the periodicity of trigonometric functions, images at 7, 173, 187, 353 degrees were the same under the polarized dark field, unless the nanorod had a significant tilt angle.

### Application to live cells

**Figure 5:**
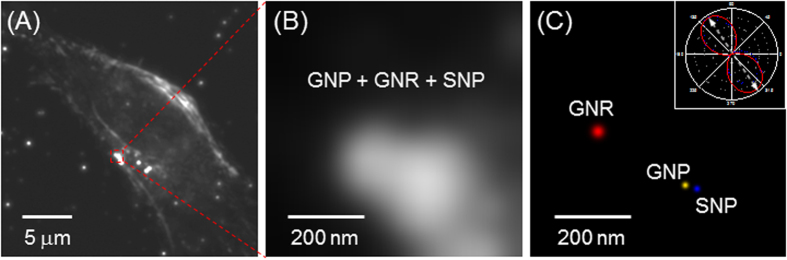
(**A** and **B**) Original EDF images of GNPs, GNRs, and SNPs in a live HeLa cell. (**C**) The reconstructed fluorescence-free super-resolution images of GNP, GNR, and SNP in the live cell and the resolved 124° orientation angle of GNR (inset).

To demonstrate the feasibility and usability of our super-resolution method, 103-nm GNPs, 80-nm SNPs, and 40-nm GNRs were naturally endocytosed by HeLa cells after incubation for 4 h. The cells were imaged using the microscope system in EDF and DIC modes (Supplementary, Fig. S10 and Movies S2 and S3). Adjacent NPs were not resolved due to diffraction limits and the complex biological environment (Fig. 5A,B). However, by applying the wavelength-modulation super-resolution technique, adjacent NPs with sub-diffraction limit distances were resolved (Fig. 5C). In addition, the orientation angle of the GNR was resolved as 124° (Fig. 5C inset).

## Discussion

Based on the wavelength-modulation EDF, super-resolution of fluorescence-free plasmonic NPs was achieved. Band-pass filters were used to modulate EDF illumination images of GNPs, GNRs, and SNPs based on their specific LSPR scattering wavelengths. By fitting their PSF with a 2D Gaussian function via a least-squares criterion algorithm, the coordinates (*x, y*) of each nanoparticle were resolved. The measured localization precisions of GNP, GNR, and SNP were 2.5 nm, 5.0 nm, and 2.9 nm, respectively, which were consistent with the CRLB and Mie theory. Finally, the super-resolution image was reconstructed based on the resolved coordinates of the NPs and the corresponding localization precisions. Furthermore, according to the spontaneous polarization of GNP scattering, the orientation angle of the GNR in 2D was resolved to provide more elaborate localization information. Subsequently, localization precisions of NPs at sub-diffraction limit resolution were applied to live cells using this novel fluorescence-free super-resolution microscopic method. Even though the LSPR scattering of plasmonic NPs were highly relying on the properties of the surrounding environment, such as refractive index of the medium and surface modifications, essentially, all these factors were only acting in the frequency domain of the scattering, i.e. inducing the scattering spectral shift. In the spatial domain (particles’ position) and time domain (detection period), these factors were negligible. Therefore, this wavelength-modulation based super-resolution method was working well regardless of homogeneous or heterogeneous environment. Although larger numbers of NPs with different materials and/or sizes would be needed to easily apply this process to more complicated biological systems or samples, this novel technique demonstrated the potential of super-resolution microscopy with fluorescence-free material. This method should be applicable to sub-cellular structure mapping after simple modifications. When the distance between individual NPs is much smaller than the particle sizes, the plasmonic coupling effect of the particles cannot be neglected, which will cause large localization errors. In this particular case, the plasmonic coupling effect should be considered to obtain better sub-diffraction limit resolution (i.e., less than a few nm).

## Methods

### Reagents and materials

The 103-nm GNP, citrate-capped 40-nm GNR, and 80-nm SNP colloidal solutions were purchased from Nanopartz^™^ (Salt Lake City, UT, USA) and BBI Life Sciences (Cardiff, UK). Cell culture medium was prepared using Dulbecco’s modified Eagle’s medium (DMEM, pH 7.4, GIBCO, Gaithersburg, MD, USA) containing 10% fetal bovine serum (GIBCO) and 1× antibiotic-antimitotic (GIBCO). Dulbecco’s phosphate buffered saline (DPBS, GIBCO, pH 7.4) was used as a cell wash buffer.

### Electron microscopy images

The true features of GNP, GNR, and SNP were observed with an environmental scanning electron microscope (ESEM, Quanta FEG 650, FEI Company) with an accelerating voltage of 30 kV. The samples for SEM imaging were prepared by dropping a small portion of the sample suspended in distilled water onto a silicon wafer and then drying the wafer in a desiccator.

5 μL suspension of the nanoparticles mixture (GNP, GNR, and SNP) was dropped onto a Cu-grid (carbon coated, 200-mesh, Ted Pella, Inc., Redding, CA, USA) and imaged by transmission electron microscopy (TEM) (2100F, JEOL Ltd, Tokyo, Japan) after the sample was completely dried. The Cu-grid was then placed on a microscope slide, immersed with protective fingernail polish and covered with a cover glass for EDF imaging^26^.

### Optical properties of GNP, GNR, and SNP

UV-Visible spectra of aqueous dispersions of GNP, GNR, and SNP were measured using a UV-Visible spectrometer (MultiSpec-1501, Shimadzu, Tokyo, Japan). Absorption peaks for GNP, GNR, and SNP were at 577 nm, 663 nm, and 477 nm, respectively.

### Lab-built microscopy system of wavelength-modulation enhanced dark-field illumination

A lab-built EDF illumination microscopy system (Supplementary, Fig. S11) was built on an Olympus BX-51 upright microscope (BX-51, Olympus, Tokyo, Japan) equipped with a CytoViva EDF illumination device (CytoViva Inc., Auburn, AL, USA) and a 100× objective lens (UPLANFLN, adjustable N.A., from 0.6 to 1.3, Olympus, Tokyo, Japan). An electron multiple charge-coupled device (EMCCD) camera (QuantEM, 512 SC, Photometrics, AZ, USA) and a color Nikon D3S digital camera (Tokyo, Japan) were installed on top of the microscope to simultaneously detect single-particle images. Band-pass filters of various wavelengths (473 ± 10 nm, 575 ± 15 nm, and 680 ± 10 nm) purchased from Semrock (Rochester, NY, USA) were used to modulate the detected scattering wavelength of the specimen. A 360° rotation analyzer (U-AN360P-2, Olympus) was installed before the cameras to analyze the orientation angle of the GNR. MetaMorph (Version 7.0, Universal Imaging, Sunnyvale, CA, USA), ThunderSTORM plug-in of ImageJ (NIH)^44^, and Origin (OriginLab) programs were used for image acquisition and data processing.

### Localization precision measurement

499 EDF images of the same single NP at the specific scattering wavelength were acquired. The particle center in each frame was analyzed using the ThunderSTORM plug-in of ImageJ. The localization precision was calculated using the following equations:


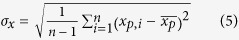



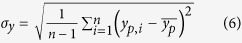



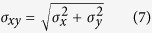


### Cell incubation with NPs

Cultures of HeLa cells were plated as previously described^45^, and were grown in cell culture medium. Cells were maintained in plastic tissue culture dishes (BD Biosciences, Bedford, MA, USA) at 37 °C with a humidified atmosphere containing 5% CO_2_. For single-cell imaging, cells were placed in a 22 mm × 22 mm cover-glass (No. 1, Deckglaser, Freiburg, Germany) and were incubated for 24 h. Adherent cells were rinsed twice with DPBS, and were then added immediately to the medium containing NPs, followed by incubation for 4 h before being used in experiments. Cover-glasses with adherent cells were washed 3 times with DPBS to remove excess NPs and were then placed under the objective lens to obtain images.

## Additional Information

**How to cite this article**: Zhang, P. *et al*. Super-resolution of fluorescence-free plasmonic nanoparticles using enhanced dark-field illumination based on wavelength-modulation. *Sci. Rep*. **5**, 11447; doi: 10.1038/srep11447 (2015).

## Supplementary Material

Supplementary Information

Supplementary Movie S1

Supplementary Movie S2

Supplementary Movie S3
