# Supplementary material for: Super-resolution of fluorescence-free plasmonic nanoparticles using enhanced dark-field illumination based on wavelength-modulation
Source: Sci Rep. 2015 Jun 15;5:11447. doi: 10.1038/srep11447 (PMC4466792; doi:10.1038/srep11447)
Supplement: Supplementary Information [file srep11447-s1.pdf]

Supplementary Information

Super-resolution of fluorescence-free plasmonic nanoparticles using enhanced dark-field illumination based on wavelength-modulation

Peng Zhang[1], Seungah Lee[2], Hyunung Yu[3], Ning Fang[4], Seong Ho Kang[1,2,*]

[1]Department of Chemistry, Graduate School, Kyung Hee University, Yongin-si, Gyeonggi-do 446-701, Korea

[2]Department of Applied Chemistry and Institute of Natural Sciences, Kyung Hee University, Yongin-si, Gyeonggi-do 446-701, Korea

[3]Center for Nanometrology, Korea Research Institute of Standards and Science, Daejeon 305-340, Korea

[4]Ames Laboratory-US Department of Energy and Department of Chemistry, Iowa State University, Ames, Iowa 50011, USA

***Corresponding author.**

Tel.: +82 31 201 3349. Fax: +82 31 201 2340. *E-mail:* shkang@khu.ac.kr (S. H. Kang).

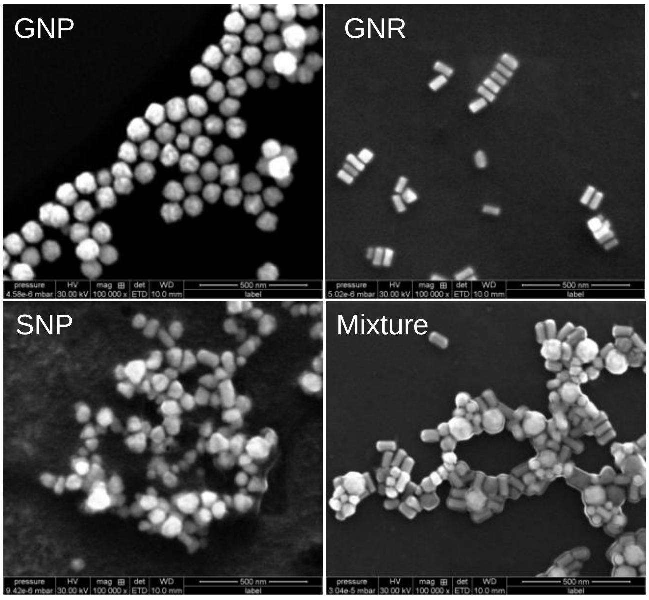

Figure S1. Scanning electron microscopy (SEM) images of GNP, GNR, SNP, and the particle mixture on a glass plate. SEM, Quanta FEG 650, FEI Co. HV, 30 kV.

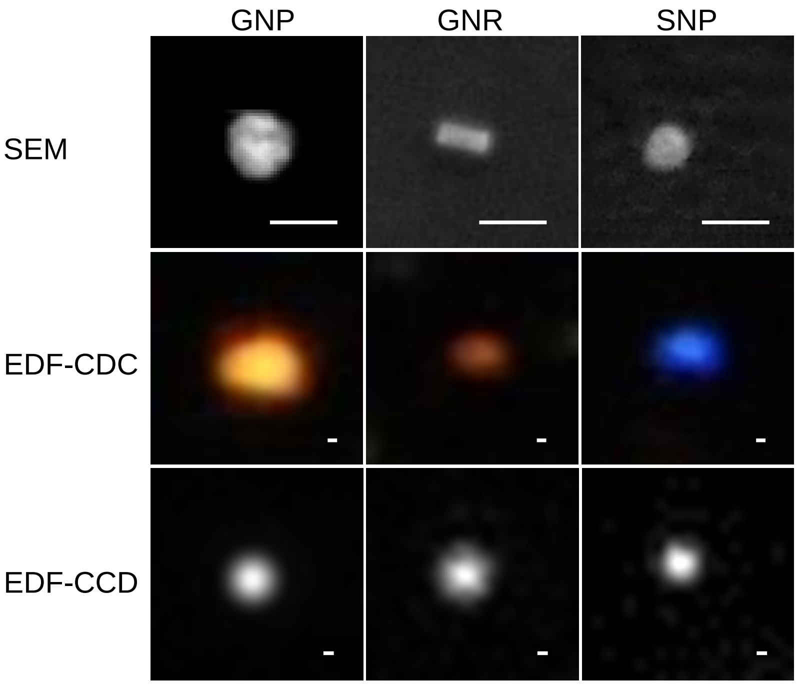

Figure S2. Comparison of SEM and optical microscopy images of GNP, GNR, and SNP. The scale bars represent 100 nm.

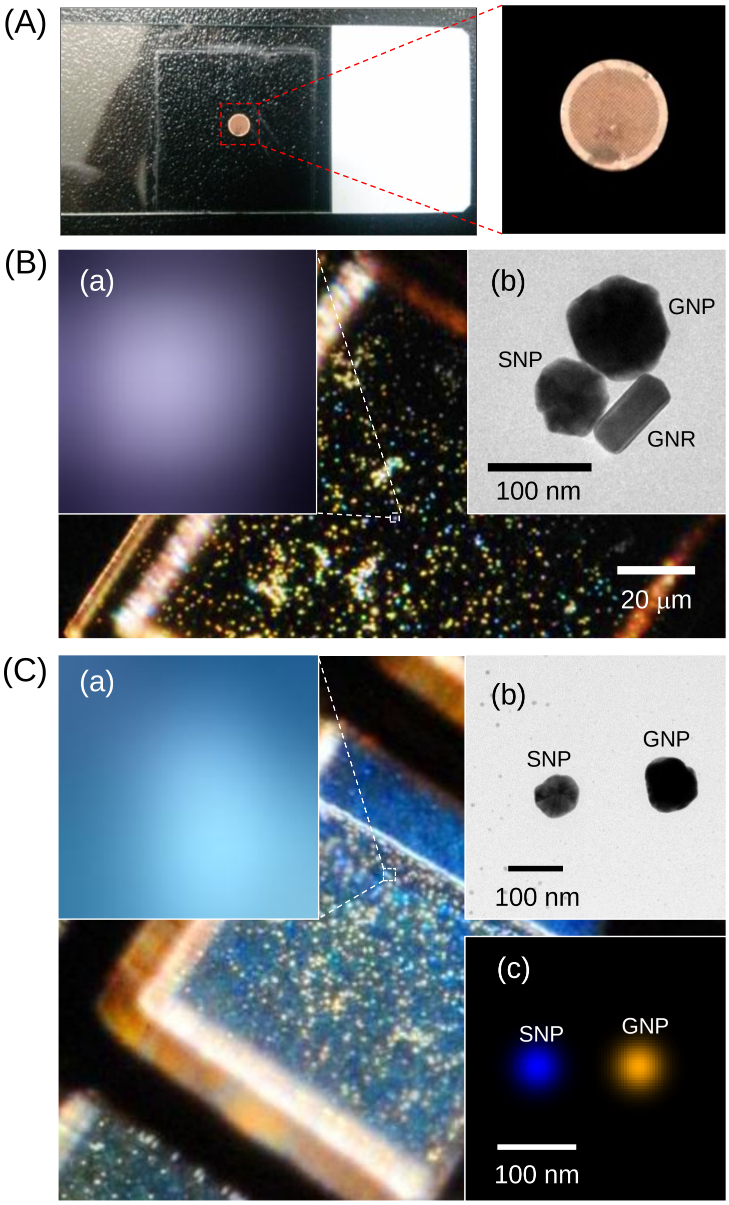

Figure S3. (A) A Cu-grid on a glass plate for the EDF and transmission electron microscopy (TEM) images of GNP, GNR, and SNP. (B) The EDF (a) and TEM images (b) of same area which contains GNP, GNR, and SNP. (C) The EDF (a), TEM (b), and reconstructed sub-diffraction (c) images of same area which contains GNP and SNP. The mixture of GNP, GNR, and SNP solution was dropped on the Cu-grid and detected by TEM and EDF microscopy.

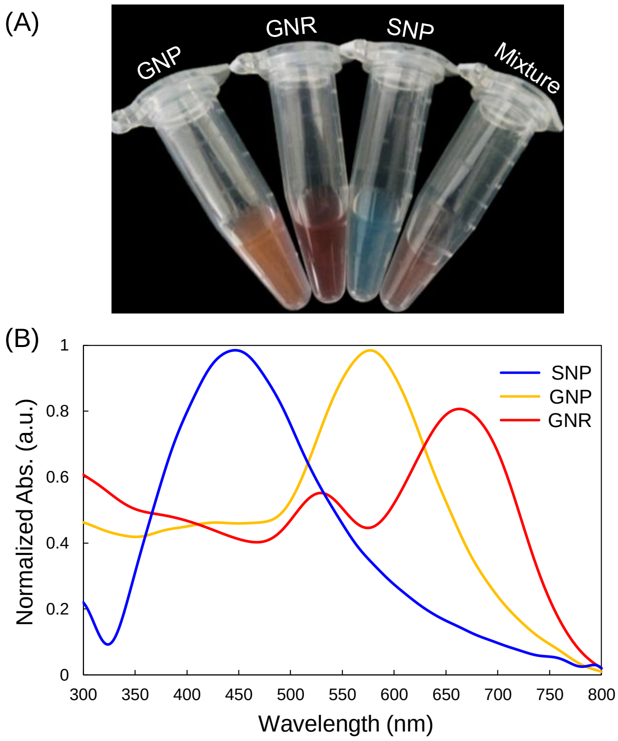

Figure S4. (A) Pictures of GNP, GNR, and SNP solutions. (B) UV-Visible absorption spectra of each NP. The absorption peaks for GNP, GNR, and SNP are 577 nm, 663 nm, and 477 nm, respectively.

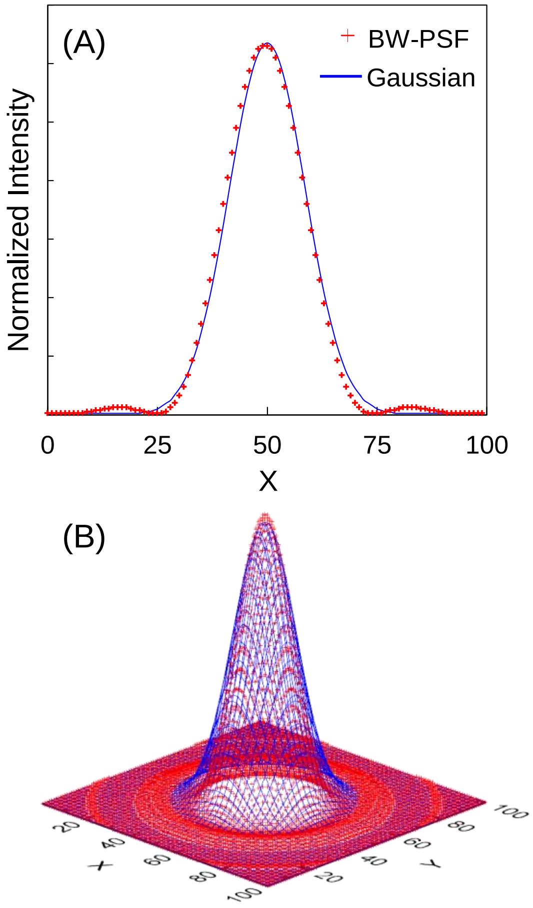

Figure S5. Comparison of Born-Wolf model based point spread function (BW-PSF) and its Gaussian approximation in 1D (A) and 2D (B).

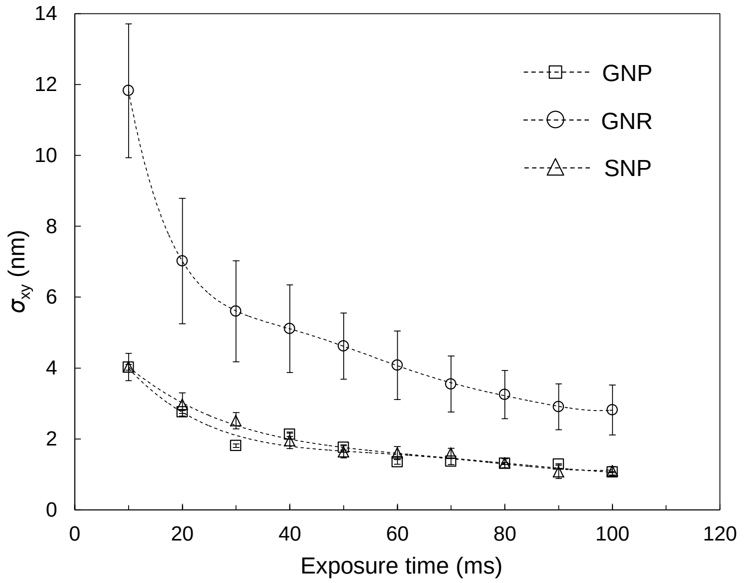

Figure S6. Localization precisions (σ_{xy}) of GNP, GNR, and SNP with various CCD camera exposure times. The error bars represent mean ± standard deviation (n = 3).

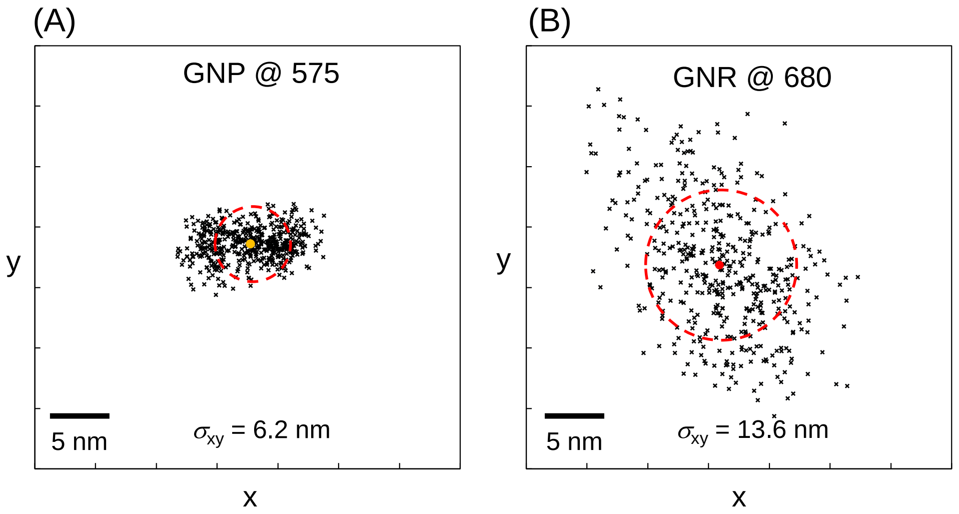

Figure S7. Typical localization precisions (σ_{xy}) of adjacent GNP (A) and GNR (B).

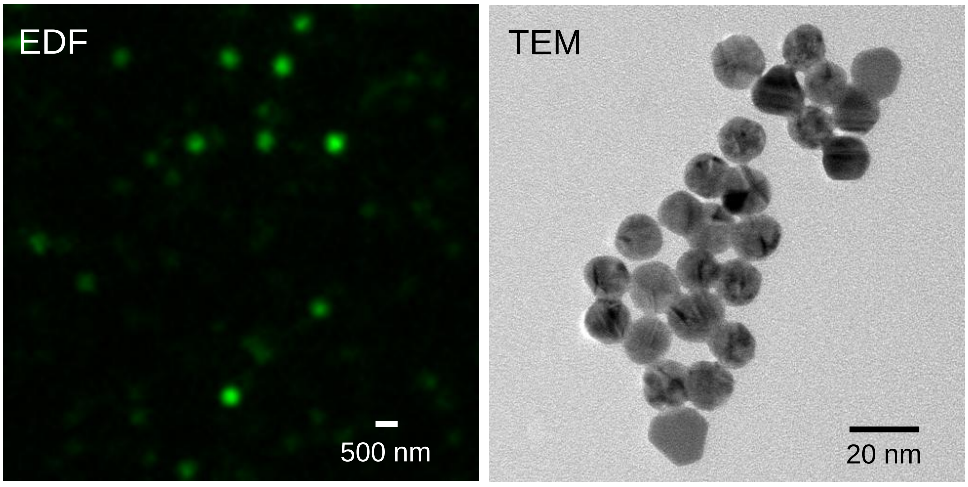

Figure S8. Typical EDF and TEM images of 12-nm GNP.

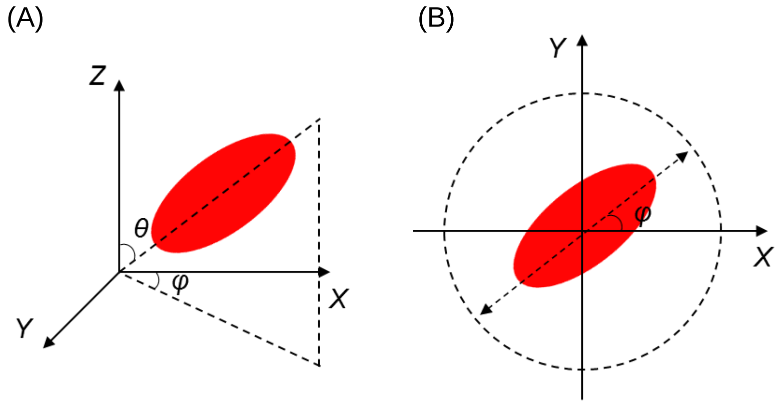

Figure S9. (A) Orientation of GNR in 3D and (B) a nanorod "lying" on the *x*-*y* surface. Here, θ is the polar angle of the GNR, which was measured from a fixed zenith direction, and φ is the orientation (azimuth) angle on the *x*-*y* plane, which was measured from the *x* direction to its orthogonal projection on the *x*-*y* plane.

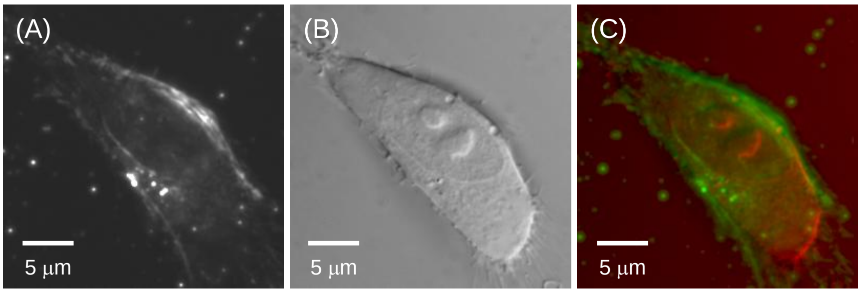

Figure S10. (A) EDF, (B) DIC and (C) merged images of NP mixtures in a living HeLa cell. The experimental conditions were the same as shown in Figure 5.

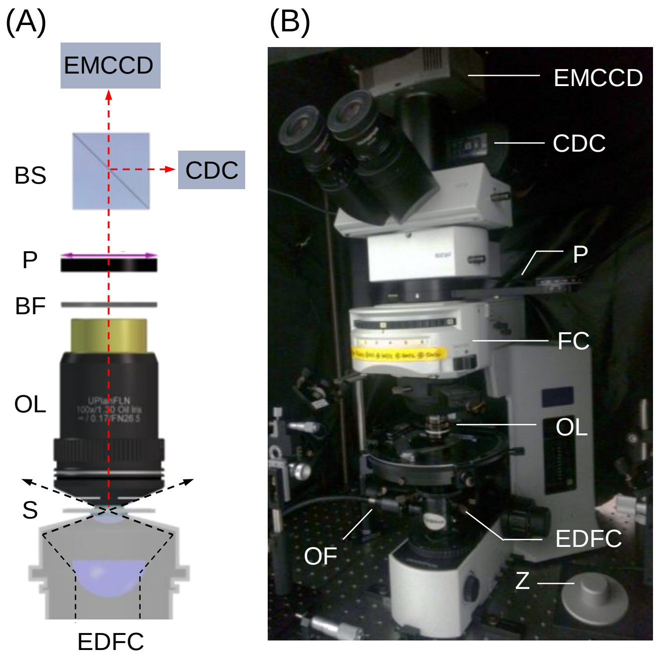

Figure S11. (A) Schematic drawing and (B) physical setup of lab-built microscopy system with wavelength-modulation enhanced dark-field illumination. Indicators: EMCCD, electron multiple charge-coupled device; CDC, colored digital camera; BS, beam splitter; P, polarizer; BF, band-pass filter; FC, filter cube; OL, objective lens; S, specimen; EDFC, enhanced dark-field condenser; OF, optical fiber; z, z-motor.

Movie S1: Orientation angle-dependent twinkle of GNRs by EDF illumination. The analyzer was rotated clockwise from 0° to 360°.

Movie S2: EDF images of a live HeLa cell with GNPs, GNRs, and SNPs.

Movie S3: DIC images of a live HeLa cell with GNPs, GNRs, and SNPs.
